# An aggregated dataset of serial morbidity and titer measurements from influenza A virus-infected ferrets

**DOI:** 10.1038/s41597-024-03256-6

**Published:** 2024-05-17

**Authors:** Troy J. Kieran, Xiangjie Sun, Hannah M. Creager, Terrence M. Tumpey, Taronna R. Maines, Jessica A. Belser

**Affiliations:** 1 Influenza Division, Centers for Disease Control and Prevention, Atlanta, GA USA; 2 Present Address: University of Pittsburgh, University of Pittsburgh Medical Center, Pittsburgh, Pennsylvania USA

**Keywords:** Influenza virus, Viral pathogenesis

## Abstract

Data from influenza A virus (IAV) infected ferrets provides invaluable information towards the study of novel and emerging viruses that pose a threat to human health. This gold standard model can recapitulate many clinical signs of infection present in IAV-infected humans, support virus replication of human, avian, swine, and other zoonotic strains without prior adaptation, and permit evaluation of virus transmissibility by multiple modes. While ferrets have been employed in risk assessment settings for >20 years, results from this work are typically reported in discrete stand-alone publications, making aggregation of raw data from this work over time nearly impossible. Here, we describe a dataset of 728 ferrets inoculated with 126 unique IAV, conducted by a single research group under a uniform experimental protocol. This collection of morbidity, mortality, and viral titer data represents the largest publicly available dataset to date of *in vivo*-generated IAV infection outcomes on a per-ferret level.

## Background & Summary

Ferrets (*Mustela putorius furo*) can become productively infected with human, avian, swine, and other zoonotic influenza A viruses (IAV) without prior host adaptation, and can display many clinical signs of disease similar to humans post-inoculation. Unlike mice or guinea pigs, ferrets permit the coincident study of both viral pathogenicity and transmissibility^1^, making them a valuable small mammalian model for public health risk assessment activities. For these reasons, data from the ferret model is considered in risk assessment rubrics by the Centers for Disease Control and Prevention (CDC) and World Health Organization (WHO)^2,3^, and the ferret is widely employed in laboratories worldwide to study the capacity of novel and emerging IAV to cause disease and transmit between animals^4^.

Despite the widespread use of ferrets in public health settings, heterogeneity across all scales (from laboratory-specific protocols and reagents, to experimental designs, to caging and facilities differences) can make comparison of results from different institutions a challenge, and preclude easy aggregation of data from multiple research groups^5,6^. To eschew these potential confounders when comparing results obtained from *in vivo* studies with different IAV, one solution is for individual research groups to make publicly available aggregated historical data that has been uniformly collected and analyzed under a consistent protocol. However, most research groups have not conducted a sufficient historical breadth of work under uniform conditions to assemble a truly diverse dataset, and/or do not have the personnel or resources to dedicate to this time-intensive effort. As such, efforts to aggregate and analyze large sets of data from IAV-inoculated ferrets are relatively few^7,8^.

Here, we present a dataset of aggregated serial data from 728 ferrets inoculated with 126 unique wild-type IAV. This dataset includes peak morbidity measurements collected during the observation period (weight loss, temperature), survival outcomes, infectious virus titers from serially collected nasal wash specimens throughout the acute phase of infection, and transmission outcomes from respiratory droplet transmission experiments. These data have supported analyses in several prior studies assessing statistical correlations present in viral titer and morbidity data^7,9^, variability in normalized and non-normalized data collected for risk assessment purposes^10^, and predictive machine learning evaluations. Collectively, this effort contributes to transparency in sharing data collected for public health risk assessment purposes, and provides an example for other research groups with uniformly collected historical data to follow a similar path.

## Methods

### Dataset availability

Data available at data.cdc.gov^11^.

### Biosafety and ethics statement

All work with infectious IAV was conducted at either biosafety level 2 or biosafety level 3 containment, including enhancements, as required by the U.S. Department of Agriculture and the Federal Select Agent Program^12^. All animal work was conducted under the guidance of the CDC’s Institutional Animal and Care and Use Committee and in an Association for Assessment and Accreditation of Laboratory Animal Care International-accredited animal facility. References to prior publications from which these records were aggregated have been compiled previously^9^.

### Influenza A viruses

126 influenza A viruses (inclusive of H1, H2, H3, H5, H7, and H9 virus subtypes) were used to inoculate ferrets. Virus stocks were propagated in either 10–11 day old embryonated hen’s eggs or Madin Darby Canine Kidney (MDCK) cells as previously described^13^.

### Ferret inoculation and sample collection

All ferrets in this work were male, between 5–12 months of age, and serologically negative to influenza A and B viruses currently circulating at the time of inoculation. Ferrets were inoculated with a high dose (10^5^–10^7^ infectious units) of virus intranasally in a 1 ml volume. A minimum of 3 ferrets were inoculated with each unique IAV. Ferrets were housed for all experiments using a HEPA-filtered Duo-Flo BioClean mobile environmental enclosure (Lab Products) for the duration of each experiment. For virus inoculation, nasal wash collection, and euthanasia procedures, ferrets were anesthetized with an intramuscular (i.m.) injection (0.2–0.5 ml) of a ketamine cocktail (25 mg/kg Ketamine, 2 mg/kg Xylazine, with or without 0.05 mg/kg Atropine) in the hamstring.

Ferrets were observed daily for clinical signs of infection for 14 days. Daily weight measurements were collected with a scale. Daily temperature readings were collected using a subcutaneous temperature transponder, 14 mm × 2 mm in size (IPTT-300, BMDS, Seaford, DE), inserted into the dorsal space between the scapulae. Experimental endpoints warranting humane euthanasia were based primarily on severe weight loss (losing >25% of pre-inoculation body weight) or onset of neurological symptoms (including hind limb weakness and/or torticollis).

Nasal wash (NW) specimens were collected on alternate days post-inoculation (p.i.), starting either day 1 or day 2 p.i.; NW specimens were collected until infectious virus was no longer detected (through day 9 p.i.), or until a ferret reached a humane endpoint. NW specimens were obtained by introducing a 1 ml volume of PBS into the nasal passages of anesthetized ferrets to induce sneezing, and collecting the aspirate using a sterile plastic dish. Aspirate was stored at −80 °C until titration. NW specimens were titered in either 10–11 day old embryonated hen’s eggs (to determine a 50% Egg Infectious Dose titer, EID_50_) or MDCK cells (to determine a Plaque Forming Units titer, PFU) as previously described^13^. All viral titers are presented as log_10_ titer and reported per milliliter. Titration limits of detection were 10^1.5^ EID_50_/ml or 10 PFU per milliliter.

For determination of transmission by respiratory droplets, a serologically naïve ferret was placed in an adjacent cage to a virus-inoculated ferret 24 hours p.i.; cages had perforated side walls which permit air exchange in the absence of direct or indirect contact (e.g. via contact with infectious virus present on fomites in the housing environment) between animals^14^. A transmission event was defined as the contact animal having detectable infectious virus in at least one NW specimen (between days 1–11 post-contact) and seroconverting to homologous virus at the end of the experiment (approx. 21 days post-contact).

## Data Records

The dataset is available at data.cdc.gov^11^.

### Ferret

Unique observational ID for each individual animal in the dataset. All ferrets were sourced from Triple F Farms (Sayre, PA, USA) unless otherwise specified in the literature^15^.

### Virus

Unique identifier for each virus used to inoculate ferrets in the dataset.

### inoc_dose

Numeric column that indicates the log_10_ infectious units used to intranasally inoculate the ferret (in a 1 ml total volume, with virus diluted in PBS).

### units

Binary column that indicates if log_10_ titers reported in NW columns are reported as EID_50_ (titration in eggs, EID) or PFU (titration in cells, PFU). EID and PFU units are not interchangeable; see usage notes for limitations in drawing comparisons between viral titers derived from different units.

### expt

Binary column that indicates if experimental results within the row are limited to virus-inoculated animals only (path) or if respiratory droplet transmissibility to a contact animal was concurrently assessed and reported within the row (RD).

### lethal

Binary column that indicates if the ferret survived the 14 day p.i. inoculation period (FALSE) or was humanely euthanized between days 1–14 p.i. due to reaching experimental endpoints (TRUE).

### lethal_day

Numerical column that indicates the day p.i. an animal was euthanized if humane endpoint criteria were reached (animals surviving the infection are reflected as 0).

### NW_typical

Binary column that indicates if every-other-day NW sample collection started on day 1 p.i. (TRUE) or day 2 p.i. (FALSE).

### RD_trans

Binary column that indicates (when a respiratory droplet transmission assessment was conducted) if virus transmission was observed based on the criteria described in the Methods (TRUE) or not (FALSE).

### HPAI

Binary column that indicates if the inoculating virus has been classified as a highly pathogenic avian influenza (HPAI) virus based on criteria of the intravenous pathogenicity index^16^ (TRUE) or is a low pathogenic avian influenza (LPAI) or non-avian virus (FALSE).

### HPAI_MBAA

Binary column that indicates if the inoculating virus is a HPAI virus that includes a multibasic amino acid HA cleavage site (TRUE) or is a HPAI virus that does not include this molecular feature or is not identified as a HPAI virus (FALSE).

### HA

Categorical column indicating hemagglutinin subtype of the inoculating virus.

### NA

Categorical column indicating neuraminidase subtype of the inoculating virus.

### Origin

Categorical column indicating the host origin of the inoculating virus. Avian, isolated from avian host or originated from an avian species during a zoonotic spillover to humans; human, human origin; swine, swine origin; canine, canine origin; variant, human infection with swine-origin virus.

### wt_loss

Numerical column that specifies the maximum percentage weight loss (normalized from preinoculation baseline weight) recorded in the inoculated animal between days 1–14 p.i. (minimum weight decrease was 1 g prior to normalization^10^; animals with no recorded drop in normalized weight are reflected as 0).

### wt_loss_day

Numerical column that indicates the day p.i. the maximum percentage weight loss reported in *wt_loss* was detected (animals with no recorded drop in weight are reflected as 0).

### temp

Numerical column that specifies the maximum increase in degrees Celsius (normalized from preinoculation baseline temperature) recorded in the inoculated animal between days 1–14 p.i. (minimum rise was 0.1 °C over baseline temperature^10^; animals with no recorded rise in temperature are reflected as 0).

### temp_day

Numerical column that indicates the day p.i. the maximum increase in degrees Celsius reported in *temp* was detected (animals with no recorded rise in temperature are reflected as 0).

### temp_5

Numerical column that specifies the maximum increase in degrees Celsius (normalized from preinoculation baseline temperature) recorded in the inoculated animal between days 1–5 p.i. (animals with no recorded rise in temperature are reflected as 0).

### temp_5_day

Numerical column that indicates the day p.i. the maximum increase in degrees Celsius reported in *temp_5* was detected (animals with no recorded rise in temperature are reflected as 0).

### d1_inoc:d9_inoc

Numerical columns reporting NW log_10_ titer/ml collected from the inoculated ferret, in the units specified in *units*. Blank spaces indicate no NW was collected that day (due to every-other-day sampling schedule, or because the ferret was euthanized due to reaching humane endpoints for the study). Day is identified in the d# format (i.e. d1 is day 1 titer).

## Technical Validation

Experimental methodology employed to collect and report morbidity and titer measurements are in agreement with other laboratories in the field employing the ferret model to perform risk assessment studies of novel and emerging influenza A viruses^6^. Technical validation of aggregated data have been confirmed and reported in prior reports^7,9,10^.

## Usage Notes

The aggregated dataset^11^ described here has numerous advantages. Consistency among ferret source, housing, experimental procedures, and titration methodology represents the greatest strength of this dataset, as it avoids confounders present when aggregating data across multiple institutions^6^. The diversity and general well-balanced distribution of virus subtypes included in this dataset permits greater extrapolation of results towards novel and emerging viruses than prior studies which have examined aggregated data with limited viral diversity across strains^8^. Furthermore, inclusion of records on a per-animal basis (and not a summary per-virus basis) strengthens the statistical power of conducted analyses while concurrently acknowledging the level of variability present between age- and sex-matched outbred ferrets inoculated with the same virus.

That said, the strengths of this dataset nonetheless contribute to several limitations. Inclusion of two titration methodologies for NW specimens is an unavoidable reality of conducting concurrent work with human IAV (which are typically preferentially propagated in mammalian cells, and may not replicate to high titer in eggs) and avian-origin IAV (which often achieve higher infectious titers following egg passage relative to mammalian cell culture) due to diverse sialic acid receptor binding specificity^17^; as such, there is a general bias in this dataset of human and variant IAV titered in eggs, and avian IAV titered in eggs. Titer vs titer comparisons may be drawn independent of the units reported here, but titer vs non-titer comparisons must be stratified by either EID or PFU as units are not interchangeable^7^. Experiments were conducted over >20 years by many laboratorians; to minimize potential confounding by subjective biases, we did not include lethargy assessments in this dataset, and restricted morbidity parameters to temperate and weight loss for which quantifiable data measurements were collected. Lethality reported is all-cause and inclusive of multiple endpoint criteria and potentially subjective assessments. The dataset includes only male ferrets, limiting investigation of sex differences in response to IAV infection^18^.

Datasets such as this permit exciting avenues of investigation, but results from these analyses should be interpreted with caution. For data science researchers who do not conduct *in vivo* work themselves, collaboration with groups who do have extensive hands-on experience and perspective in this area is strongly recommended. Extrapolation of results from ferrets to humans should similarly proceed with caution. All ferrets in this dataset were serologically naïve to seasonally circulating influenza viruses prior to use and the inoculation methodology employed (a high dose in a large volume) is meant to assess the pathogenic capacity of the virus examined, but does not represent a realistic immunologic background as the general human population and does not emulate a physiologically relevant exposure route or dose.

## Data Availability

Records in the dataset do not require additional code for subsequent analysis.
